# A Case Report of Sarcoidosis Mimicking Vertebral Metastasis

**DOI:** 10.1155/2018/5326324

**Published:** 2018-09-09

**Authors:** Anchalia Chandrakumaran, Henry R. Bateman, Rehan Qayyum

**Affiliations:** ^1^Department of Internal Medicine, Virginia Commonwealth University Health Systems, Richmond, VA, USA; ^2^Department of Pathology, Virginia Commonwealth University Health Systems, Richmond, VA, USA

## Abstract

A 35-year-old African American male, previously healthy, presented with lower back and bilateral lower extremity pain associated with intermitted night sweats and weight loss. Imaging was concerning diffuse vertebral metastatic lesions. He underwent extensive workup to evaluate for metastatic disease. However, right iliac crest, mediastinal, and left inguinal lymph node biopsies were consistent with sarcoidosis. He was started on methotrexate, folic acid, and prednisone.

## 1. Introduction

Sarcoidosis is a multisystem granulomatous disorder that is characterized by the formation of non-necrotizing granulomas in various organs including the lungs and lymphatic system. Usually, it is a diagnosis of exclusion due to its complexity and heterogeneous multisystem involvement [1]. African Americans have a threefold higher age-adjusted annual incidence of sarcoidosis compared with Caucasians [2]. However, bone involvement in sarcoidosis is rare (1–13%), and involvement of the vertebrae is even rarer [3]. We report a case of a young African American male who presented with bilateral lower extremity pain and was found to have several vertebral lesions concerning diffuse metastatic disease. However, biopsies were consistent with sarcoidosis. This case illustrates the utility of several imaging modalities and laboratory testing such as serum calcium levels, serum angiotensin converting enzyme levels, and histopathology in distinguishing metastatic disease from sarcoidosis.

## 2. Case Presentation

A previously healthy 35-year-old African American male presented with a one-month history of worsening lower back and bilateral lower extremity pain, intermittent night sweats, and 32 kg unintentional weight loss over the course of a year. He did not have saddle anesthesia or urinary or fecal incontinence. He was initially seen in a primary care clinic and was diagnosed with sciatica. As symptoms continued to worsen, he underwent a computed tomography (CT) scan of the lumbar scan as an outpatient that was concerning osseous spinal metastasis. He was started on prednisone 10 mg daily and was referred to the oncology clinic at our center. Prednisone gave him minimal symptomatic relief. While waiting to be seen in the oncology clinic, the patient had an episode of leg weakness with near-fall prompting him to present to the emergency department of our hospital and was admitted for further evaluation. His vital signs were stable. He had no palpable cervical, supraclavicular, axillary, or inguinal lymph nodes. Neurological exam was normal with intact strength and sensation in both lower extremities.

**Figure 1:**
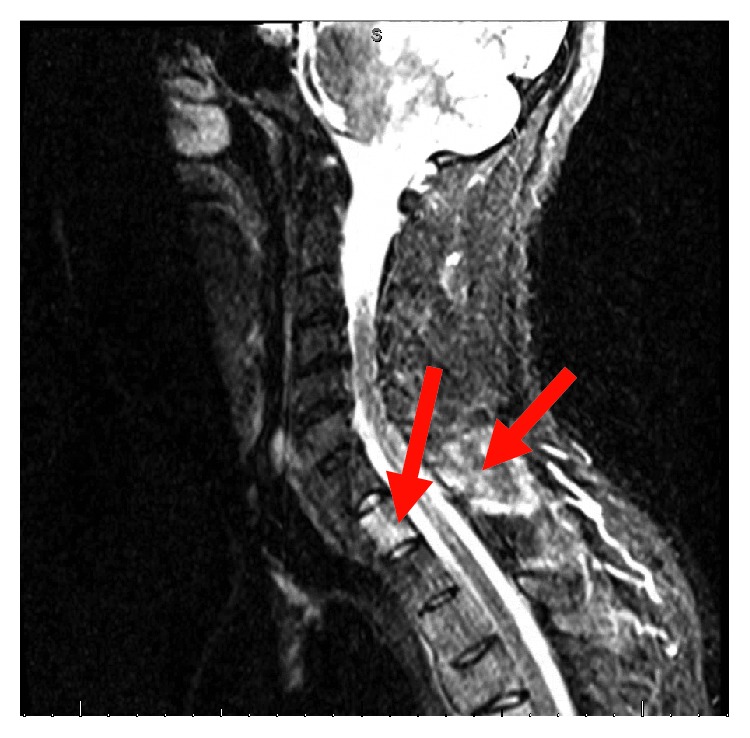
T2 sagittal MRI cervical and lumbar spine.

**Figure 2:**
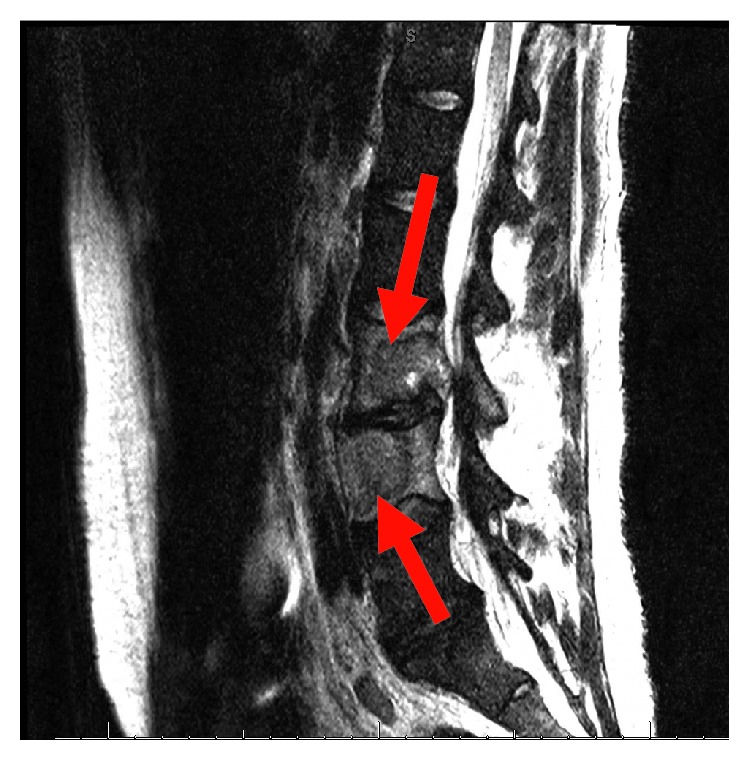
T2 sagittal MRI lumbar spine.

**Figure 3:**
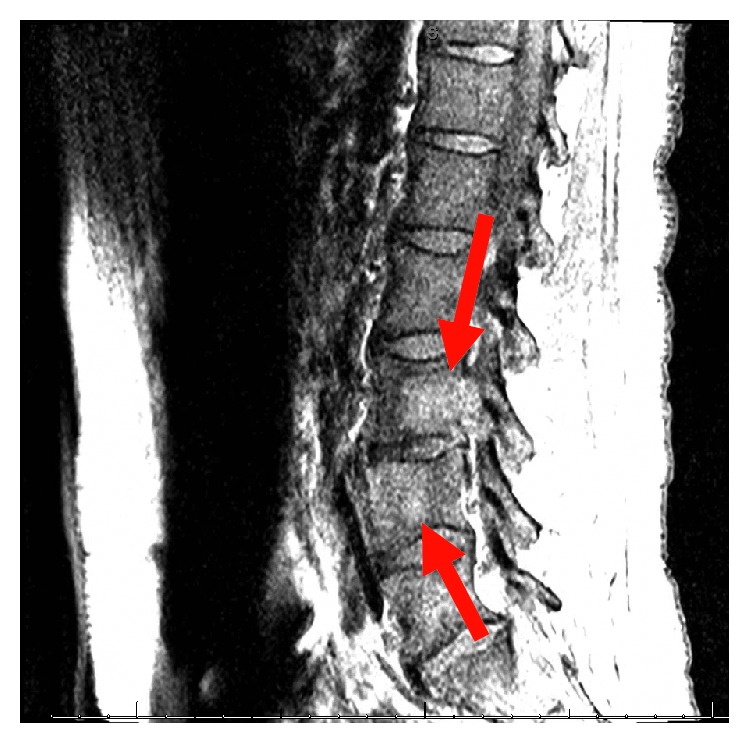
T1 sagittal segment MRI lumbar spine.

**Figure 4:**
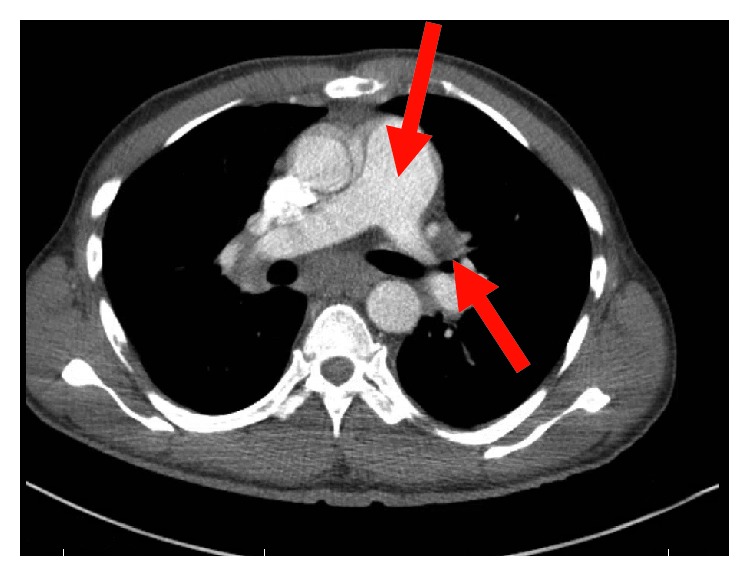
CT chest with contrast showing enlarged pulmonary artery, mediastinal, and hilar lymphadenopathy.

**Figure 5:**
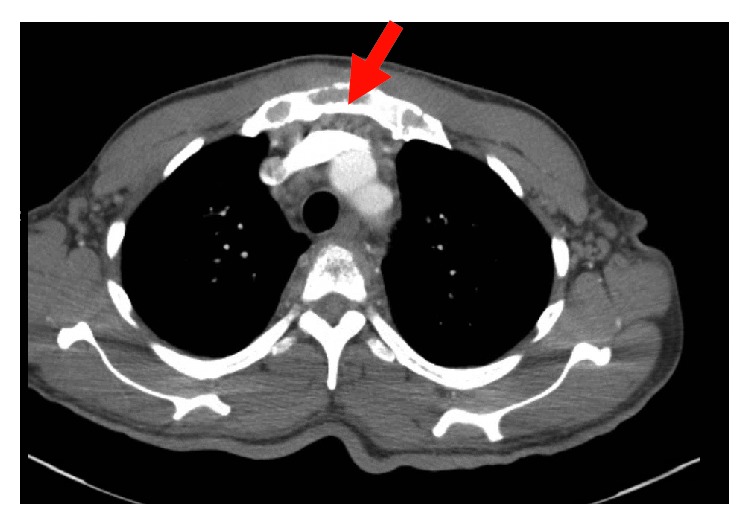
CT chest with contrast showing lesions in the ribs and sternum.

**Figure 6:**
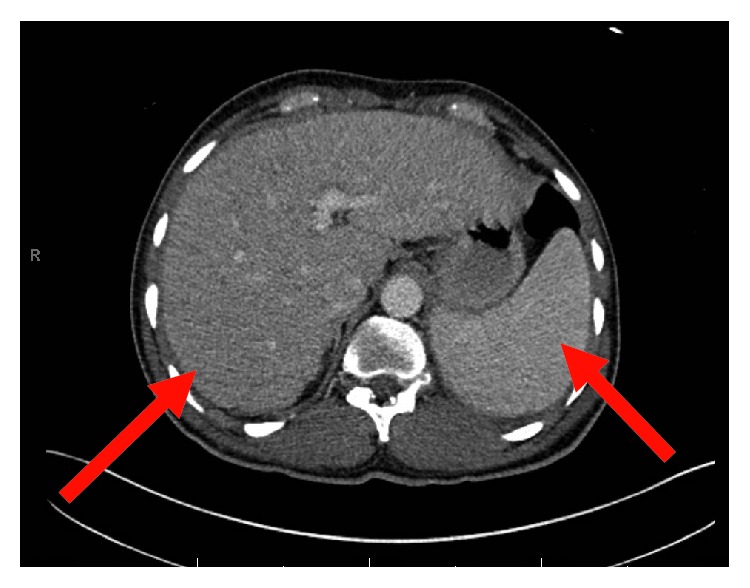
CT abdomen/pelvis showing hepatosplenomegaly.

His complete blood count and serum electrolytes were normal including a normal serum calcium level at 8.1 mg/dL. He tested negative for human immunodeficiency virus 1 and 2 antibodies. Magnetic resonance imaging (MRI) of the cervical, thoracic, and lumbar spine showed several enhancing lesions in T11, T12, L3, L4 vertebral bodies, right sacrum, and ilium that were concerning metastatic disease. There was effacement of the right lateral recess and right neural foramen at the L3-L4 and effacement of the left lateral recess and left neural foramen at the L4-L5 due to tumor retropulsion (Figures 1–3). In addition, a small epidural tumor was noted at the T5 vertebral level without significant spinal canal stenosis or cord compression. Imaging was also concerning osseous metastasis involving the sternum and multiple ribs. Incidentally, narrowing of the neural foramen at left T2-T3 and right C7-T1 and T5-T6 levels was also noted. Since the findings were concerning diffuse metastatic disease, a CT scan of the chest, abdomen, and pelvis were performed and showed bilateral hilar and mediastinal adenopathy, mild cardiomegaly, and dilated main pulmonary artery measuring 3.6 cm (Figures 4 and 5). Enlarged liver measuring 18.1 cm, enlarged spleen measuring 12.4 cm, and multiple bilateral enlarged pelvic sidewall, external iliac, and inguinal lymph nodes concerning lymphoma or metastatic disease are shown in Figure 6. Ultrasound of the scrotum did not reveal any testicular masses.

**Figure 7:**
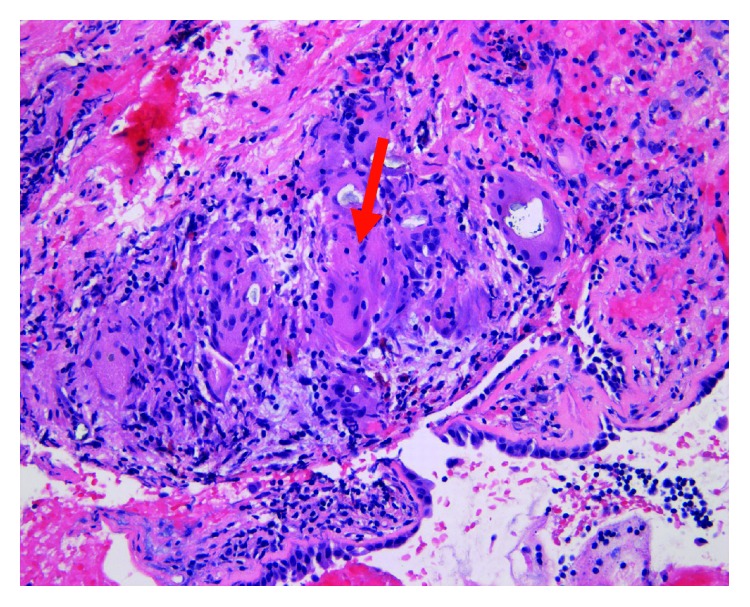
Fragments of respiratory epithelium with foreign body giant cells.

**Figure 8:**
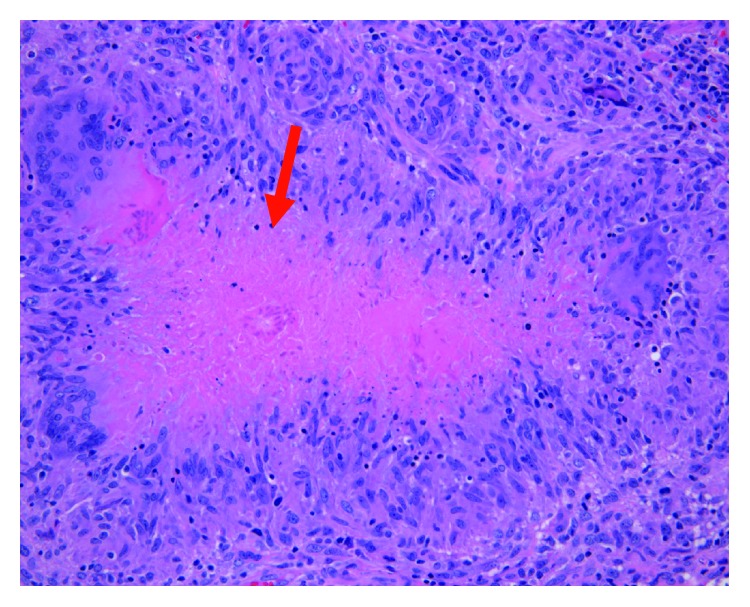
Left inguinal lymph node biopsy with necrotizing granuloma.

**Figure 9:**
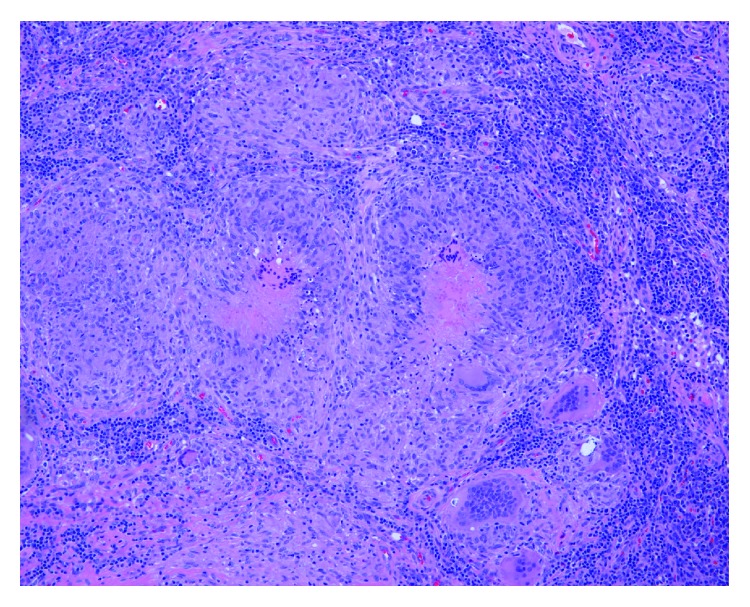
Left inguinal lymph node biopsy demonstrating necrotizing and non-necrotizing granulomas.

He underwent extensive screening for hematologic and solid tumor malignancies including serum protein electrophoresis, urine immunofixation, beta-human chorionic gonadotrophin hormone levels, and fecal occult blood test that were all negative. He subsequently underwent a CT-guided core needle biopsy of the left iliac crest lesion that was significant for noncaseating and necrotizing granulomas. Histochemical stains for Grocott's methenamine silver (GMS) and Ziehl-Neelsen stains were negative for fungal elements and acid-fast bacilli, respectively. Due to high suspicion of malignancy, he also underwent an endoscopic bronchial ultrasound with transbronchial needle aspiration of the inferior mediastinal lymph node which found non-necrotizing granulomas but did not reveal any malignant cells (Figures 7–9). Fungal culture and acid-fast bacilli culture from the transbronchial aspirate were again negative. Serum ACE level was 62 U/L (normal 14–82 U/L).

**Figure 10:**
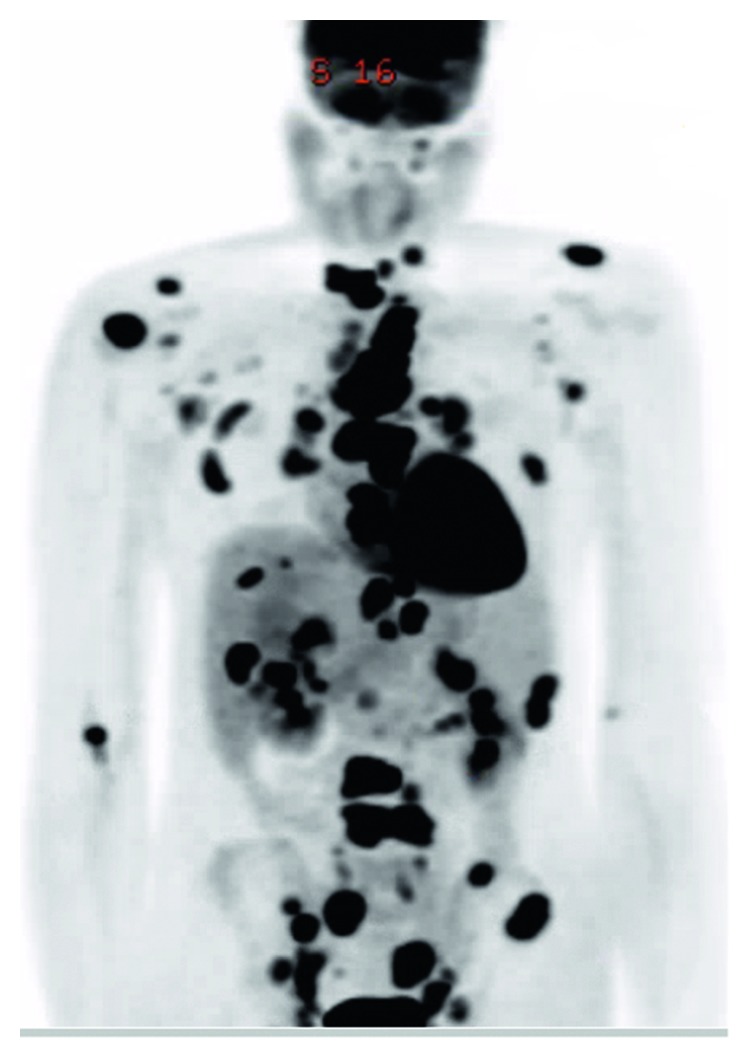
FDG-PET scan extensive hypermetabolic osseous and nodal disease.

Neurosurgery was consulted, and they did not recommend any acute neurosurgical intervention. The patient was discharged with follow-up in pulmonology clinic. Since there was concern that his steroid therapy prior to admission could have masked lymphoma, he had a left inguinal node excisional biopsy, a month later, that showed necrotizing and non-necrotizing granulomatous lymphadenopathy and was negative for acid-fast or fungal microorganisms. Since there was concern for a process with high metabolic activity, he also had an ^18^F-labeled fluorodeoxyglucose (^18^F-FDG) positron electron topography (PET) scan that was significant for extensive hypermetabolic osseous and nodal disease (Figure 10).

## 3. Differential Diagnosis

The patient's initial presentation with significant weight loss, bilateral lower extremity weakness, lower back pain, and imaging were concerning malignancy especially non-Hodgkin's lymphoma. However, the patient's young age and lack of primary tumor on imaging was less concerning a malignant etiology. His clinical symptoms, lack of fever, and systemic signs did not support an infectious process. Pyogenic osteomyelitis was ruled out with negative biopsy results. Furthermore, he did not have a history of immunosuppression, imprisonment, or travel to an endemic area that would put place him at a risk for fungal or *Mycobacterium tuberculosis* infection. Castleman's disease could also present with lymphadenopathy, hepatosplenomegaly, and pulmonary findings but would typically have a plasmablastic histopathology [4]. Although sarcoidosis was considered, given the extent of spinal lesions and lack of pulmonary symptoms, it was lower in the differential diagnoses list, and the more life-threatening etiologies needed to be excluded. Left iliac crest biopsy, transbronchial needle aspiration of the mediastinal lymph node, and left inguinal lymph node biopsy showed that non-necrotizing granulomas were consistent with a diagnosis of sarcoidosis.

## 4. Treatment and Follow-Up

The patient was started on prednisone 60 mg daily, methotrexate 15 mg weekly, and folic acid 1 mg daily. Two weeks after starting methotrexate, he presented to the emergency department with headaches, worsening weakness, and back pain. Repeat MRI of his cervical, thoracic, and lumbar spine was unchanged from his prior imaging. Lumbar puncture ruled out meningitis. He was started on 400 mg infliximab (planned for a course of 0, 2, and 6 weeks and then every 6 weeks) in addition to methotrexate and prednisone. He was evaluated by a neurologist for his headaches, and it was thought to be likely secondary to muscle spasms. Topiramate 50 mg daily and sumatriptan 100 mg daily as needed were initiated for his headaches. His pain was managed with oxycodone 20 mg every 6 hours as needed and extended release morphine every 8 hours until his sarcoidosis is under better control. He continues to follow-up closely with rheumatology and neurology as an outpatient.

## 5. Discussion

Vertebral sarcoidosis is extremely rare with spinal sarcoidosis occurring in <1% of patients with sarcoidosis [5]. Differentiating osseous sarcoidosis from metastatic lesions based on MRI can be extremely challenging even by experienced radiologists. Moore et al. evaluated several imaging features to discriminate metastatic bone lesions and sarcoidosis including peri- or intralesional fat, specific border characteristics, the presence of extraosseous soft-tissue mass, and lesions with posterior element involvement. However, none of these features reliably distinguished the two disease processes [6]. The lymphadenopathy, hepatosplenomegaly, and bone lesions in sarcoidosis may also mimic metastatic disease.

In addition, osseous sarcoidosis may show increased uptake on the ^18^F-FDG-PET scan since sarcoidosis is a hypermetabolic, inflammatory process and involves the activation of macrophages. Hence, ^18^F-FDG-PET scan may produce false-positive results and complicate the diagnosis of metastatic neoplastic disease. Baldini et al. [7–10] reported a case of a 71-year-old male who was diagnosed with well-differentiated carcinoid tumor and underwent a lung lobectomy but presented a month later with acute renal failure and hypercalcemia in the presence of low serum parathyroid hormone levels. The FDG-PET scan showed increased uptake in the vertebrae consistent with malignancy. However, he had elevated ACE levels, and bone marrow biopsy revealed sarcoid granulomas [11]. Thus, the value of an ^18^F-FDG-PET scan in reaching a diagnosis in this case is uncertain. In 2006, Waanders et al. presented case of an asymptomatic 61-year-old male who presented with hypercalcemia, and he was found to have extensive lesions in bones, lungs, and lymphadenopathy mimicking extensive metastatic disease. However, biopsy of left iliac crest and peripheral lung biopsy with epithelioid granulomas confirmed the diagnosis of sarcoidosis [12]. Our case and the above noted cases highlight the importance of biopsy confirmation of the bone lesions [13].

The diagnosis of sarcoidosis in our case was further complicated by normal serum calcium and ACE levels. The sensitivity, specificity, positive, and negative predictive values of ACE activity in sarcoidosis are 0.55, 0.99, 0.95, and 0.90, respectively [14]. However, a 1975 study by Lieberman measuring ACE levels in 400 patients found that the ACE level was elevated in 15 of the 17 patients with active sarcoidosis [6]. Thus, it is unusual for sarcoidosis to present with normal ACE levels.

Since vertebral sarcoidosis is a rare disease, there is very little literature on the treatment. The clinical course of the disease is variable with some case reports of improvement without treatment. Thus, there is no well-defined indication for therapy. Corticosteroids are used as first-line drugs. Other therapeutic options reported to be beneficial include methotrexate, hydroxychloroquine, cyclosporine, and tumor necrosis factor-alpha (TNF-alpha) inhibitors [9, 13, 15]. Methotrexate is the most commonly used second-line agent in the treatment of sarcoidosis, and the drug is associated with leukopenia and pulmonary and hepatic toxicity. However the prevalence of methotrexate-induced hepatic and pulmonary toxicity is low in sarcoidosis [16]. Methotrexate can be used in conjunction with corticosteroids to enable reduced steroid dose. Since TNF-alpha have been implicated in granuloma formation, TNF-alpha inhibitors have been increasingly used to successfully treat refractory sarcoidosis [15].

## 6. Learning Points/Take-Home Messages

- Osseous sarcoidosis lesions cannot be reliably distinguished from metastatic lesions on routine MRI studies even by readers experienced in assessing these lesions
- Osseous sarcoidosis may show increased uptake on ^18^F-FDG-PET and may lead to false-positive results.
- It is extremely important to obtain a biopsy in evaluating multifocal bone lesions
- Sarcoidosis can present with normal ACE levels
- No specific guidelines for the treatment of osseous sarcoidosis are currently available. Corticosteroids are the first line of treatment.
